# *N*-Phenyl-2-(1,2,3,4-tetra­hydro­naph­thalen-1-yl­idene)hydrazinecarbo­thio­amide

**DOI:** 10.1107/S1600536814001585

**Published:** 2014-01-25

**Authors:** Adriano Bof de Oliveira, Bárbara Regina Santos Feitosa, Christian Näther, Inke Jess

**Affiliations:** a Departamento de Química, Universidade Federal de Sergipe, Av. Marechal Rondon s/n, Campus, 49100-000 São Cristóvão-SE, Brazil; b Institut für Anorganische Chemie, Christian-Albrechts-Universität zu Kiel, Max-Eyth Strasse 2, D-24118 Kiel, Germany

## Abstract

The conformation of the title mol­ecule, C_17_H_17_N_3_S, is stabilized by an intra­molecular N—H⋯N hydrogen bond involving the azometinic group. The dihedral angle between the two aromatic rings is 36.49 (06)°. The non-aromatic ring of the tetra­lone substituent adopts a sofa conformation. In the crystal, mol­ecules are linked by pairs of N—H⋯S hydrogen bonds related *via* centres of symmetry, forming dimers.

## Related literature   

For the synthesis and pharmacological activity of ketone­thio­semicarbazones, see: Thanigaimalai *et al.* (2011 ▶). For one of the first reports of the synthesis of thio­semicarbazone derivatives, see: Freund & Schander (1902 ▶). For the synthesis and crystal structure of 2-(1,2,3,4-tetra­hydro­naphthalen-1-yl­idene)hydrazinecarbo­thio­amide, see: de Oliveira *et al.* (2012 ▶).
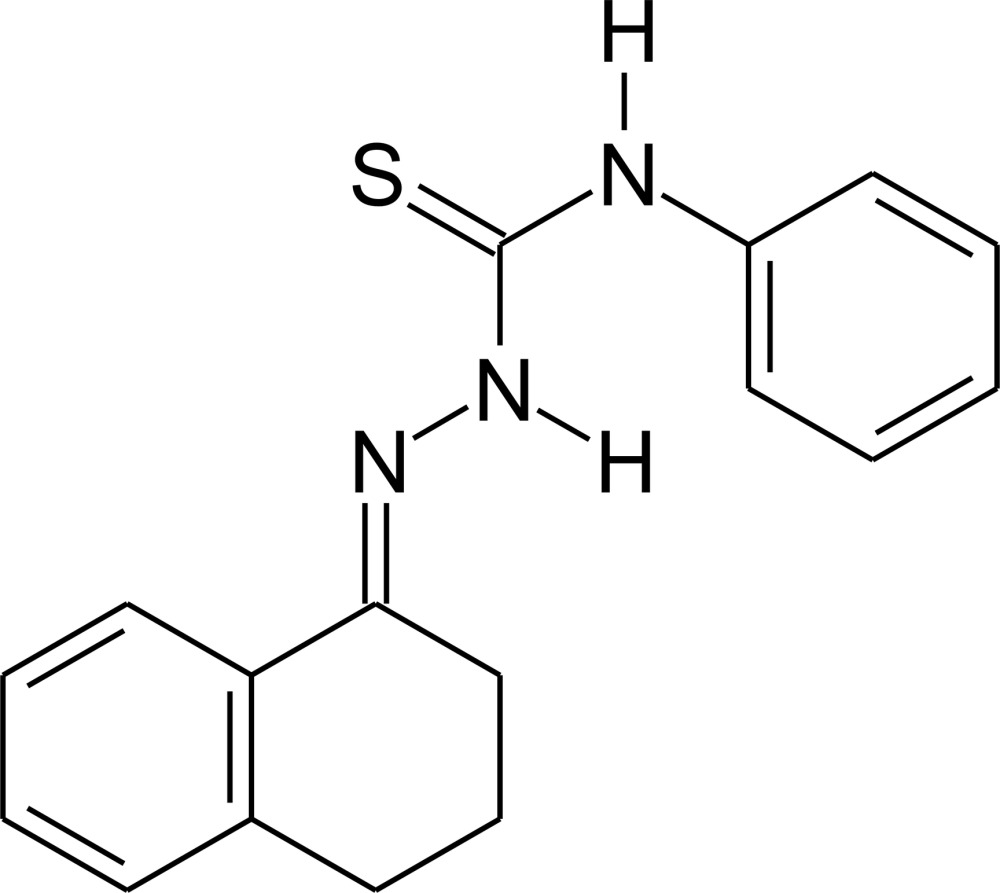


## Experimental   

### 

#### Crystal data  

C_17_H_17_N_3_S
*M* *_r_* = 295.40
Monoclinic, 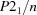

*a* = 8.4415 (3) Å
*b* = 18.0256 (7) Å
*c* = 10.0260 (3) Å
β = 107.495 (2)°
*V* = 1455.02 (9) Å^3^
*Z* = 4
Mo *K*α radiation
μ = 0.22 mm^−1^
*T* = 200 K
0.3 × 0.3 × 0.2 mm

#### Data collection  

Stoe IPDS-1 diffractometer
23139 measured reflections
3520 independent reflections
3045 reflections with *I* > 2σ(*I*)
*R*_int_ = 0.069

#### Refinement  

*R*[*F* ^2^ > 2σ(*F* ^2^)] = 0.036
*wR*(*F* ^2^) = 0.089
*S* = 1.05
3520 reflections
190 parameters
H-atom parameters constrained
Δρ_max_ = 0.26 e Å^−3^
Δρ_min_ = −0.24 e Å^−3^

### 

Data collection: *X-AREA* (Stoe & Cie, 2008 ▶); cell refinement: *X-AREA*; data reduction: *X-RED32* (Stoe & Cie, 2008 ▶); program(s) used to solve structure: *SHELXS97* (Sheldrick, 2008 ▶); program(s) used to refine structure: *SHELXL97* (Sheldrick, 2008 ▶); molecular graphics: *DIAMOND* (Brandenburg, 2006 ▶); software used to prepare material for publication: *publCIF* (Westrip, 2010 ▶).

## Supplementary Material

Crystal structure: contains datablock(s) I. DOI: 10.1107/S1600536814001585/bt6959sup1.cif

Structure factors: contains datablock(s) I. DOI: 10.1107/S1600536814001585/bt6959Isup2.hkl

Supporting information file. DOI: 10.1107/S1600536814001585/bt6959Isup3.cml

CCDC reference:

Additional supporting information:  crystallographic information; 3D view; checkCIF report

## Figures and Tables

**Table 1:** Hydrogen-bond geometry (Å, °)

*D*—H⋯*A*	*D*—H	H⋯*A*	*D*⋯*A*	*D*—H⋯*A*
N1—H1*N*⋯S1^i^	0.88	2.70	3.5793 (11)	176
N3—H2*N*⋯N2	0.88	2.12	2.5630 (15)	111

Symmetry code: (i) 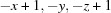.

**Fig. 1.**
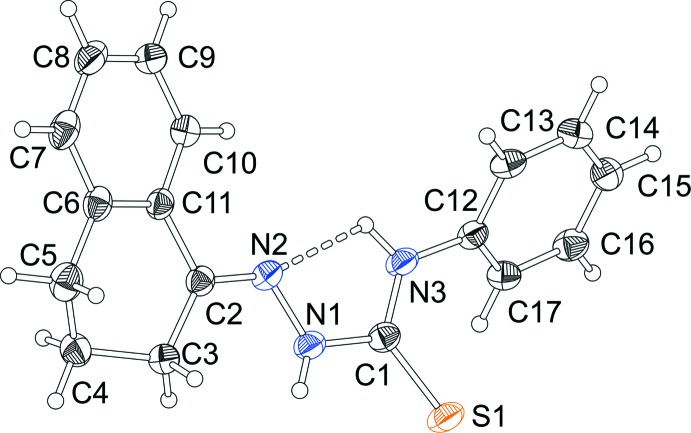
: The molecular structure of the title compound with labeling and displacement ellipsoids drawn at the 50% probability level, with intramolecular hydrogen bonding shown as dashed line.

**Fig. 2.**
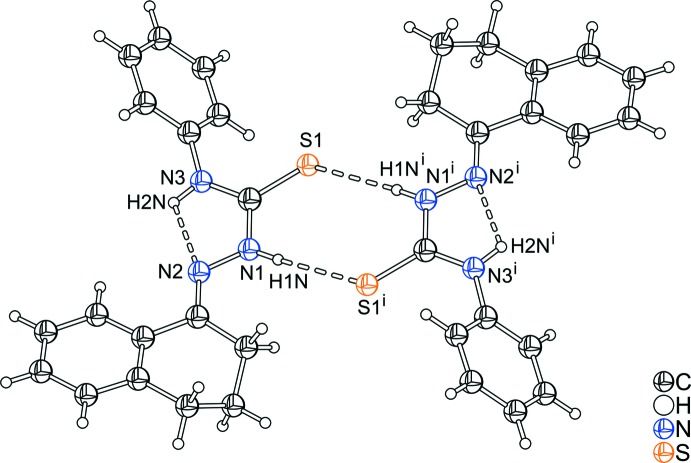
: Crystal structure of the title compound with inter- and intramolecular hydrogen bonding shown as dashed lines. Symmetry code: (i) -x + 1, -y, -z + 1.

## supplementary crystallographic information

### 1. Comment

Thiosemicarbazone derivatives have a wide range of pharmacological properties. For example, ketonethiosemicarbazones show pharmacological activity against melanogenesis in melanoma B16 cells (Thanigaimalai *et al.*, 2011). As part of our study on synthesis of thiosemicarbazone compounds, we report herein the crystal structure of a derivative of 2-(1,2,3,4-Tetrahydronaphthalen-1-ylidene)hydrazinecarbothioamide (Oliveira *et al.*, 2012). The title compound (Figure 1), in which the molecular structure matches the asymmetric unit, is not planar and the dihedral angle between the two aromatic rings is 36.49 (06)°. The maximum deviation from the mean plane of the non-H atoms for the non-aromatic ring of the tetralone substituent amount to 0.5003 (12) Å for C4, which corresponds to a sofa conformation. The molecule shows a *trans* conformation for the atoms about the N2–N1 bond. Due to an intramolecuar hydrogen bond involving the N—H···N atoms (Figure 1), a *cis* conformation about the N1–C1 bond is observed. The planarity, atoms distances and angles of the thiosemicarbazone fragment are in agreement with literature data (de Oliveira *et al.*, 2012). The molecules are connected *via* centrosymmetric pairs of N—H···S, forming dimers (Figure 1, Figure 2 and Table 1).

### 2. Experimental

Starting materials were commercially available and were used without further purification. The tetralone-thiosemicarbazone derivative synthesis was adapted from a procedure reported previously (Freund & Schander, 1902). The hydrochloric acid catalyzed reaction of tetralone (8,83 mmol) and 4-phenylthiosemicarbazide (8,83 mmol) in ethanol (50 ml) was refluxed for 6 h. After cooling and filtering, the title compound was obtained. Crystals suitable for X-ray diffraction of the title compound were obtained in tethahydrofuran by the slow evaporation of solvent.

### 3. Refinement

All H atoms were located in difference map but were positioned with idealized geometry and were refined using a riding model with *U*_iso_(H) = 1.2 *U*_eq_(C and N) with C—H = 0.95 Å for aromatic, C—H = 0.99 Å for methylene and N—H = 0.88 Å for N—H H atoms.

### Crystal data

**Table:**

C_17_H_17_N_3_S	*F*(000) = 624
*M**_r_* = 295.40	*D*_x_ = 1.348 Mg m^−^^3^
Monoclinic, *P*2_1_/*n*	Melting point: 452 K
Hall symbol: -P 2yn	Mo *K*α radiation, λ = 0.71073 Å
*a* = 8.4415 (3) Å	Cell parameters from 26099 reflections
*b* = 18.0256 (7) Å	θ = 2.3–28.1°
*c* = 10.0260 (3) Å	µ = 0.22 mm^−^^1^
β = 107.495 (2)°	*T* = 200 K
*V* = 1455.02 (9) Å^3^	Cubic, yellow
*Z* = 4	0.3 × 0.3 × 0.2 mm

### Data collection

**Table:**

Stoe IPDS-1 diffractometer	3045 reflections with *I* > 2σ(*I*)
Radiation source: fine-focus sealed tube, Stoe IPDS-1	*R*_int_ = 0.069
Graphite monochromator	θ_max_ = 28.1°, θ_min_ = 2.3°
φ scans	*h* = −11→11
23139 measured reflections	*k* = −23→23
3520 independent reflections	*l* = −13→12

### Refinement

**Table:**

Refinement on *F*^2^	Primary atom site location: structure-invariant direct methods
Least-squares matrix: full	Secondary atom site location: difference Fourier map
*R*[*F*^2^ > 2σ(*F*^2^)] = 0.036	Hydrogen site location: inferred from neighbouring sites
*wR*(*F*^2^) = 0.089	H-atom parameters constrained
*S* = 1.05	*w* = 1/[σ^2^(*F*_o_^2^) + (0.0371*P*)^2^ + 0.419*P*] where *P* = (*F*_o_^2^ + 2*F*_c_^2^)/3
3520 reflections	(Δ/σ)_max_ = 0.001
190 parameters	Δρ_max_ = 0.26 e Å^−^^3^
0 restraints	Δρ_min_ = −0.24 e Å^−^^3^

### Special details

**Table:**

Geometry. All e.s.d.'s (except the e.s.d. in the dihedral angle between two l.s. planes) are estimated using the full covariance matrix. The cell e.s.d.'s are taken into account individually in the estimation of e.s.d.'s in distances, angles and torsion angles; correlations between e.s.d.'s in cell parameters are only used when they are defined by crystal symmetry. An approximate (isotropic) treatment of cell e.s.d.'s is used for estimating e.s.d.'s involving l.s. planes.
Refinement. Refinement of *F*^2^ against ALL reflections. The weighted *R*-factor *wR* and goodness of fit *S* are based on *F*^2^, conventional *R*-factors *R* are based on *F*, with *F* set to zero for negative *F*^2^. The threshold expression of *F*^2^ > σ(*F*^2^) is used only for calculating *R*-factors(gt) *etc*. and is not relevant to the choice of reflections for refinement. *R*-factors based on *F*^2^ are statistically about twice as large as those based on *F*, and *R*- factors based on ALL data will be even larger.

### Fractional atomic coordinates and isotropic or equivalent isotropic displacement parameters (Å^2^)

**Table:**

	*x*	*y*	*z*	*U*_iso_*/*U*_eq_	
S1	0.37784 (5)	0.09351 (2)	0.54195 (3)	0.03751 (11)	
C1	0.47689 (15)	0.07366 (7)	0.70981 (13)	0.0270 (3)	
N1	0.55649 (13)	0.00734 (6)	0.74440 (11)	0.0284 (2)	
H1N	0.5703	−0.0195	0.6754	0.034*	
N2	0.65846 (13)	−0.00109 (6)	0.88038 (11)	0.0278 (2)	
C2	0.74837 (15)	−0.06010 (7)	0.91151 (12)	0.0250 (2)	
C3	0.74732 (17)	−0.12214 (7)	0.81071 (13)	0.0291 (3)	
H3A	0.8085	−0.1061	0.7455	0.035*	
H3B	0.6312	−0.1327	0.7549	0.035*	
C4	0.82614 (18)	−0.19283 (7)	0.88489 (14)	0.0328 (3)	
H4A	0.7561	−0.2134	0.9394	0.039*	
H4B	0.8337	−0.2303	0.8149	0.039*	
C5	0.99852 (18)	−0.17606 (8)	0.98201 (15)	0.0363 (3)	
H5A	1.0483	−0.2220	1.0311	0.044*	
H5B	1.0702	−0.1583	0.9265	0.044*	
C6	0.99093 (16)	−0.11786 (7)	1.08793 (14)	0.0286 (3)	
C7	1.10660 (17)	−0.11733 (8)	1.22097 (15)	0.0351 (3)	
H7	1.1929	−0.1532	1.2435	0.042*	
C8	1.09825 (18)	−0.06577 (8)	1.32042 (15)	0.0383 (3)	
H8	1.1796	−0.0657	1.4097	0.046*	
C9	0.9709 (2)	−0.01430 (8)	1.28947 (14)	0.0382 (3)	
H9	0.9627	0.0203	1.3585	0.046*	
C10	0.85514 (18)	−0.01313 (7)	1.15781 (14)	0.0332 (3)	
H10	0.7684	0.0226	1.1370	0.040*	
C11	0.86494 (15)	−0.06406 (7)	1.05523 (12)	0.0262 (3)	
N3	0.48970 (14)	0.11902 (6)	0.81878 (11)	0.0290 (2)	
H2N	0.5513	0.1028	0.9008	0.035*	
C12	0.41601 (15)	0.18985 (7)	0.81780 (12)	0.0255 (2)	
C13	0.51258 (16)	0.24662 (8)	0.89485 (13)	0.0303 (3)	
H13	0.6266	0.2382	0.9427	0.036*	
C14	0.44270 (18)	0.31549 (8)	0.90199 (14)	0.0341 (3)	
H14	0.5089	0.3542	0.9551	0.041*	
C15	0.27640 (18)	0.32813 (8)	0.83178 (14)	0.0339 (3)	
H15	0.2284	0.3754	0.8362	0.041*	
C16	0.18101 (16)	0.27136 (8)	0.75537 (14)	0.0323 (3)	
H16	0.0671	0.2799	0.7071	0.039*	
C17	0.24953 (16)	0.20221 (7)	0.74832 (13)	0.0286 (3)	
H17	0.1828	0.1634	0.6961	0.034*	

### Atomic displacement parameters (Å^2^)

**Table:**

	*U*^11^	*U*^22^	*U*^33^	*U*^12^	*U*^13^	*U*^23^
S1	0.0426 (2)	0.0436 (2)	0.02103 (15)	0.01742 (15)	0.00155 (13)	−0.00099 (13)
C1	0.0239 (6)	0.0313 (6)	0.0248 (6)	0.0019 (5)	0.0060 (5)	0.0000 (5)
N1	0.0290 (5)	0.0306 (6)	0.0223 (5)	0.0048 (4)	0.0029 (4)	−0.0006 (4)
N2	0.0277 (5)	0.0311 (6)	0.0223 (5)	0.0017 (4)	0.0040 (4)	0.0024 (4)
C2	0.0243 (6)	0.0258 (6)	0.0246 (6)	−0.0015 (5)	0.0070 (5)	0.0022 (4)
C3	0.0313 (6)	0.0284 (6)	0.0261 (6)	−0.0019 (5)	0.0066 (5)	−0.0003 (5)
C4	0.0397 (7)	0.0266 (6)	0.0331 (7)	0.0021 (5)	0.0125 (6)	−0.0002 (5)
C5	0.0343 (7)	0.0360 (7)	0.0395 (7)	0.0090 (6)	0.0127 (6)	0.0045 (6)
C6	0.0252 (6)	0.0289 (6)	0.0315 (6)	−0.0017 (5)	0.0082 (5)	0.0069 (5)
C7	0.0254 (6)	0.0386 (7)	0.0377 (7)	−0.0014 (5)	0.0038 (5)	0.0131 (6)
C8	0.0364 (7)	0.0414 (8)	0.0292 (6)	−0.0121 (6)	−0.0020 (5)	0.0101 (6)
C9	0.0509 (9)	0.0318 (7)	0.0266 (6)	−0.0076 (6)	0.0037 (6)	0.0003 (5)
C10	0.0405 (7)	0.0268 (7)	0.0288 (6)	0.0009 (5)	0.0050 (5)	0.0019 (5)
C11	0.0263 (6)	0.0255 (6)	0.0254 (6)	−0.0027 (5)	0.0058 (5)	0.0044 (4)
N3	0.0294 (5)	0.0337 (6)	0.0202 (5)	0.0072 (4)	0.0019 (4)	0.0002 (4)
C12	0.0263 (6)	0.0297 (6)	0.0205 (5)	0.0030 (5)	0.0069 (4)	0.0008 (4)
C13	0.0248 (6)	0.0391 (7)	0.0242 (6)	−0.0001 (5)	0.0033 (5)	−0.0019 (5)
C14	0.0360 (7)	0.0323 (7)	0.0310 (6)	−0.0038 (5)	0.0057 (5)	−0.0058 (5)
C15	0.0383 (7)	0.0303 (7)	0.0318 (7)	0.0055 (5)	0.0086 (6)	−0.0012 (5)
C16	0.0263 (6)	0.0388 (7)	0.0292 (6)	0.0054 (5)	0.0044 (5)	−0.0012 (5)
C17	0.0255 (6)	0.0323 (7)	0.0266 (6)	−0.0014 (5)	0.0058 (5)	−0.0036 (5)

### Geometric parameters (Å, º)

**Table:**

S1—C1	1.6773 (13)	C7—H7	0.9500
C1—N3	1.3427 (16)	C8—C9	1.383 (2)
C1—N1	1.3642 (16)	C8—H8	0.9500
N1—N2	1.3846 (14)	C9—C10	1.3864 (19)
N1—H1N	0.8800	C9—H9	0.9500
N2—C2	1.2894 (16)	C10—C11	1.3995 (18)
C2—C11	1.4823 (16)	C10—H10	0.9500
C2—C3	1.5055 (18)	N3—C12	1.4190 (16)
C3—C4	1.5238 (18)	N3—H2N	0.8800
C3—H3A	0.9900	C12—C17	1.3861 (18)
C3—H3B	0.9900	C12—C13	1.3892 (18)
C4—C5	1.518 (2)	C13—C14	1.3854 (19)
C4—H4A	0.9900	C13—H13	0.9500
C4—H4B	0.9900	C14—C15	1.387 (2)
C5—C6	1.508 (2)	C14—H14	0.9500
C5—H5A	0.9900	C15—C16	1.3826 (19)
C5—H5B	0.9900	C15—H15	0.9500
C6—C7	1.3963 (18)	C16—C17	1.3849 (19)
C6—C11	1.4033 (18)	C16—H16	0.9500
C7—C8	1.380 (2)	C17—H17	0.9500
			
N3—C1—N1	114.44 (11)	C7—C8—C9	119.65 (13)
N3—C1—S1	125.48 (10)	C7—C8—H8	120.2
N1—C1—S1	120.06 (9)	C9—C8—H8	120.2
C1—N1—N2	117.35 (10)	C8—C9—C10	120.11 (14)
C1—N1—H1N	116.9	C8—C9—H9	119.9
N2—N1—H1N	121.6	C10—C9—H9	119.9
C2—N2—N1	118.38 (11)	C9—C10—C11	120.61 (13)
N2—C2—C11	116.45 (11)	C9—C10—H10	119.7
N2—C2—C3	124.48 (11)	C11—C10—H10	119.7
C11—C2—C3	119.01 (11)	C10—C11—C6	119.32 (12)
C2—C3—C4	112.33 (10)	C10—C11—C2	120.94 (11)
C2—C3—H3A	109.1	C6—C11—C2	119.70 (11)
C4—C3—H3A	109.1	C1—N3—C12	127.91 (11)
C2—C3—H3B	109.1	C1—N3—H2N	116.0
C4—C3—H3B	109.1	C12—N3—H2N	116.0
H3A—C3—H3B	107.9	C17—C12—C13	119.87 (12)
C5—C4—C3	109.89 (11)	C17—C12—N3	121.61 (11)
C5—C4—H4A	109.7	C13—C12—N3	118.44 (11)
C3—C4—H4A	109.7	C14—C13—C12	120.06 (12)
C5—C4—H4B	109.7	C14—C13—H13	120.0
C3—C4—H4B	109.7	C12—C13—H13	120.0
H4A—C4—H4B	108.2	C13—C14—C15	120.17 (13)
C6—C5—C4	110.78 (11)	C13—C14—H14	119.9
C6—C5—H5A	109.5	C15—C14—H14	119.9
C4—C5—H5A	109.5	C16—C15—C14	119.45 (13)
C6—C5—H5B	109.5	C16—C15—H15	120.3
C4—C5—H5B	109.5	C14—C15—H15	120.3
H5A—C5—H5B	108.1	C15—C16—C17	120.79 (12)
C7—C6—C11	118.80 (13)	C15—C16—H16	119.6
C7—C6—C5	120.80 (12)	C17—C16—H16	119.6
C11—C6—C5	120.40 (12)	C16—C17—C12	119.66 (12)
C8—C7—C6	121.45 (13)	C16—C17—H17	120.2
C8—C7—H7	119.3	C12—C17—H17	120.2
C6—C7—H7	119.3		
			
N3—C1—N1—N2	−9.03 (16)	C5—C6—C11—C10	176.54 (12)
S1—C1—N1—N2	169.57 (9)	C7—C6—C11—C2	175.51 (11)
C1—N1—N2—C2	−172.78 (11)	C5—C6—C11—C2	−5.79 (18)
N1—N2—C2—C11	175.47 (10)	N2—C2—C11—C10	11.35 (18)
N1—N2—C2—C3	−1.69 (18)	C3—C2—C11—C10	−171.33 (12)
N2—C2—C3—C4	−163.68 (12)	N2—C2—C11—C6	−166.29 (11)
C11—C2—C3—C4	19.23 (16)	C3—C2—C11—C6	11.04 (17)
C2—C3—C4—C5	−53.39 (15)	N1—C1—N3—C12	−177.29 (12)
C3—C4—C5—C6	58.18 (15)	S1—C1—N3—C12	4.2 (2)
C4—C5—C6—C7	149.58 (12)	C1—N3—C12—C17	47.42 (19)
C4—C5—C6—C11	−29.09 (17)	C1—N3—C12—C13	−135.95 (14)
C11—C6—C7—C8	0.67 (19)	C17—C12—C13—C14	−0.13 (19)
C5—C6—C7—C8	−178.02 (13)	N3—C12—C13—C14	−176.81 (12)
C6—C7—C8—C9	1.4 (2)	C12—C13—C14—C15	−0.3 (2)
C7—C8—C9—C10	−1.9 (2)	C13—C14—C15—C16	0.3 (2)
C8—C9—C10—C11	0.4 (2)	C14—C15—C16—C17	0.1 (2)
C9—C10—C11—C6	1.6 (2)	C15—C16—C17—C12	−0.5 (2)
C9—C10—C11—C2	−175.99 (12)	C13—C12—C17—C16	0.51 (19)
C7—C6—C11—C10	−2.16 (18)	N3—C12—C17—C16	177.09 (11)

### Hydrogen-bond geometry (Å, º)

**Table:**

*D*—H···*A*	*D*—H	H···*A*	*D*···*A*	*D*—H···*A*
N1—H1*N*···S1^i^	0.88	2.70	3.5793 (11)	176
N3—H2*N*···N2	0.88	2.12	2.5630 (15)	111

Symmetry code: (i) −*x*+1, −*y*, −*z*+1.
